# Supplementary material for: Laser-Induced Interdigital Structured Graphene Electrodes Based Flexible Micro-Supercapacitor for Efficient Peak Energy Storage
Source: Molecules. 2022 Jan 5;27(1):329. doi: 10.3390/molecules27010329 (PMC8746467; doi:10.3390/molecules27010329)
Supplement: Supplementary file 1 [file molecules-27-00329-s001.zip › molecules-1501454-SI.pdf]

Supplementary Materials

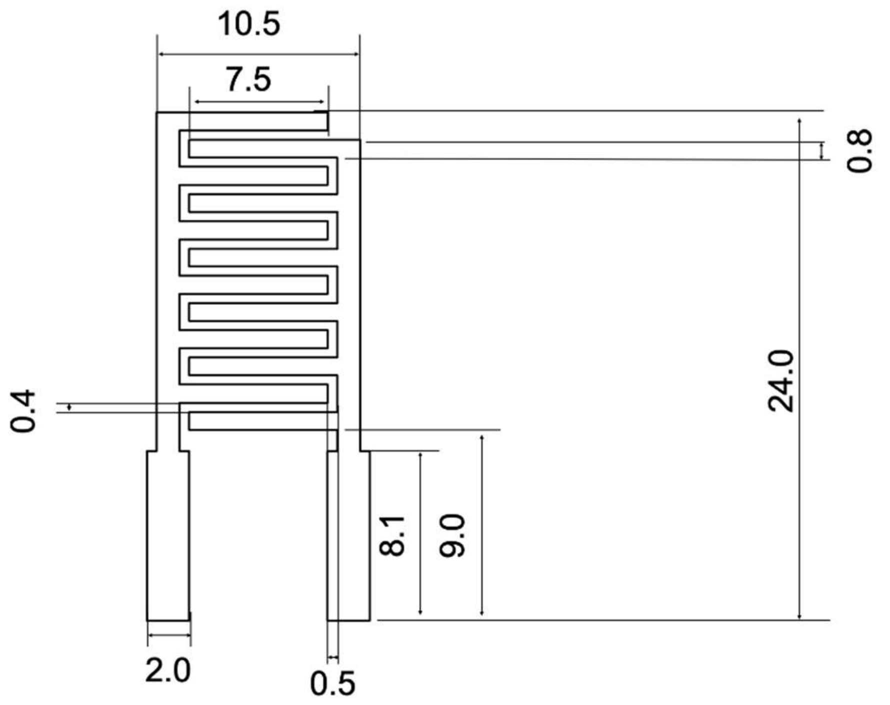

Figure S1. Details of the technical drawing of the template of the applied IMSC cell geometry (unit in mm).

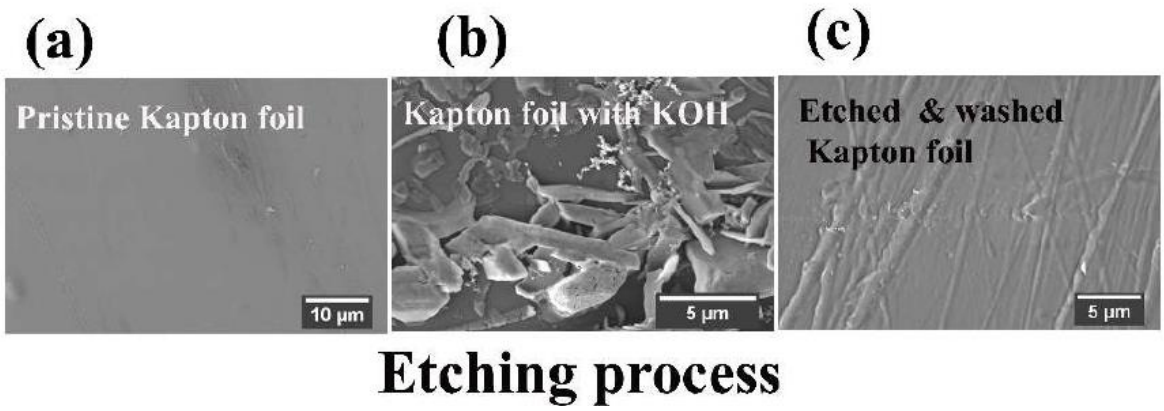

Figure S2. SEM images of the surface changing due to the KOH etching process of PI-foil: (**a**) pristine PI foil, (**b**) PI foil with dried KOH and (**c**) after etching and washing process of PI-foil.

Table S1: Some values calculated from GCD curves of this LIG-MSC device.

Current Density (mA/cm^2)	Discharge time (s)	Using Eq. (2-4) Areal capacitance C_A (mF/cm^2)	Areal Energy density E_A (μWh/cm^2)	Areal Energy density P_A (mW/cm^2)	Using Eq. (6-8) Sp. capacitance C_m (mF/g)	Sp. Energy density E_m (mWh/kg)	Sp. Power density P_m (W/kg)
**0.03**	**30.75**	**0.461**	**0.256**	**0.029**	**166.1**	**92.25**	**10.81**
**0.05**	**17.86**	**0.446**	**0.248**	**0.049**	**160.7**	**89.28**	**17.99**
**0.07**	**12.04**	**0.421**	**0.234**	**0.069**	**151.7**	**84.27**	**25.19**
**0.10**	**7.66**	**0.383**	**0.212**	**0.110**	**137.8**	**76.56**	**35.97**

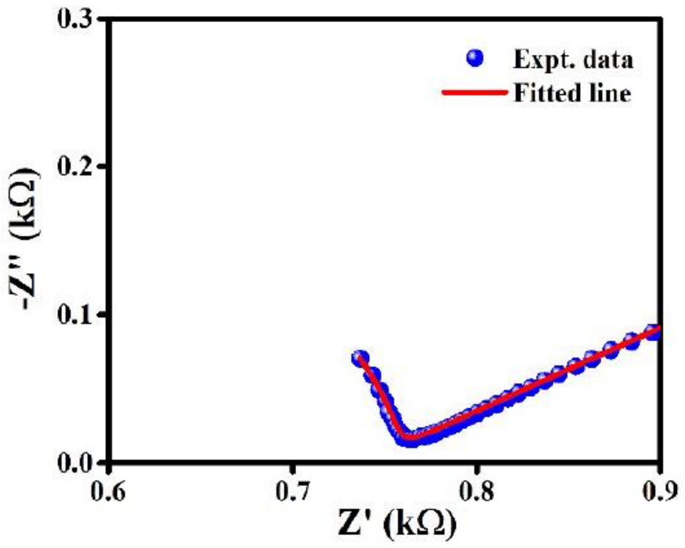

Figure S3. EIS image at high frequency region of this LIG-MSC device.
